# Analysis of the brain transcriptome in lines of laying hens divergently selected for feather pecking

**DOI:** 10.1186/s12864-020-07002-1

**Published:** 2020-08-27

**Authors:** Clemens Falker-Gieske, Andrea Mott, Siegfried Preuß, Sören Franzenburg, Werner Bessei, Jörn Bennewitz, Jens Tetens

**Affiliations:** 1 Department of Animal Sciences, Georg-August-University, Burckhardtweg 2, 37077 Göttingen, Germany; 2 Institute of Animal Science, University of Hohenheim, Garbenstr. 17, 70599 Stuttgart, Germany; 3 Institute of Clinical Molecular Biology, Christian-Albrechts-University of Kiel, Rosalind-Franklin-Straße 12, 24105 Kiel, Germany; 4 Center for Integrated Breeding Research, Georg-August-University, Albrecht-Thaer-Weg 3, 37075 Göttingen, Germany

**Keywords:** Feather pecking, Circadian clock, Brain transcriptome, Chicken, RNA-sequencing

## Abstract

**Background:**

Feather pecking (FP) in laying hens reduces animal welfare and leads to economic losses for the layer industry. FP is considered a heritable condition that is influenced by dysregulation of neurotransmitter homeostasis, the gut microbiome, and the immune system. To identify genes and biological pathways responsible for FP behavior we compared the brain transcriptomes of 48 hens divergently selected for FP. In addition, we tested if high feather peckers (HFP) and low feather peckers (LFP) respond differently to light since light has been shown to trigger FP behavior.

**Results:**

Of approximately 48 million reads/sample an average of 98.4% were mapped to the chicken genome (GRCg6a). We found 13,070 expressed genes in the analyzed brains of which 423 showed differential expression between HFP and LFP. Genes of uncertain function and non-coding RNAs were overrepresented among those transcripts. Functional analyses revealed the involvement of cholinergic signaling, postsynaptic activity, membrane channels, and the immune system. After the light stimulus, 28 genes were found to be differentially expressed. These included an interaction cluster of core components of the circadian clock. However, differences in the response to light between HFP and LFP were not detectable.

**Conclusions:**

Genes involved in cholinergic signaling, channel activity, synaptic transmission, and immune response were found to be involved in FP behavior. We propose a model in which the gut microbiota modulates the immune system, which in turn affects cholinergic signaling. This might have an influence on monoamine signaling with possible involvement of GABA or glutamate signaling.

## Background

Feather pecking (FP) is a serious problem of the layer industry causing economic losses and massive impairments of animal welfare. The propensity to perform this damaging behavior is a complex trait influenced by numerous environmental factors as well as a genetic component. Reported heritability estimates of around 0.15 [1–3] indicate the possibility of genetic selection against FP. Despite intensive research during the last decades, the underlying mechanisms are still not well understood.

A longstanding theory explained FP as a redirected foraging behavior [4, 5], which was not confirmed in later studies. Instead, it has been linked to general locomotor activity [6, 7] and feather eating [7, 8]. With respect to the latter, it was reported that the inclusion of feathers in the diet affects the chicken’s gut microbiome [9] as well as the actual pecking behavior [10]. Comparisons of the cecal microbiomes between layer lines divergently selected for FP or with different actual FP behavior revealed apparent differences [11–13]. It is difficult to determine, whether these differences are a cause for FP or just a consequence of feather consumption. The hypothesis of a link with the gut microbiome is, however, convincing as FP is influenced by the serotonergic system [14–16], which comprises a central and a peripheral part [17]. Central serotonin partly controls dopamine and thus also affects reward-related behaviors. The peripheral part is mainly found in enterochromaffin cells of the gastrointestinal tract. Both systems do interact via the bloodstream or the vagus nerve [17]. Gut microbiota can affect the serotonergic system in various ways such as the activation of the immune system [17, 18]. The latter is notable as FP has also been directly linked to immune response [19, 20]. Comprehensive genomic and transcriptomic studies might help to bring ends together by identifying potentially involved genes and pathways but are still rare. Several gene expression studies linked FP to genes involved in serotonergic signaling, either directly [21, 22] or indirectly via dopaminergic [22] or GABAergic [21] signaling or via a link to obsessive-compulsive disorders [23]. Mapping studies identified loci connecting FP to dopaminergic [24], GABAergic [25] and serotonergic [2, 24, 26] signaling as well as to the immune system [26].

As comprehensively reviewed by [18], it thus seems likely that peripheral serotonin, the gut microbiome, and the immune system are interacting with central serotonin and dopamine to influence FP. Despite the huge body of evidence for the involvement of the mentioned mechanisms in FP, it is still not understood in detail how they interact. With the aim to gain further insight into the underlying mechanisms, we used RNA-sequencing (RNA-seq) to analyze the whole brain transcriptomes of 24 full-sib pairs of hens from two layer lines divergently selected for FP behavior. As light intensity is known to trigger the actual behavior given the respective motivation [27, 28], we not only compared transcriptomes between lines but also before and after light stimulation. To our knowledge, this is the first comprehensive brain transcriptome study using RNA-seq with respect to feather pecking in layers. Our key results point to an involvement of cholinergic signaling and a contribution of the immune system, which complements with previous studies and might be a step towards an integrated model for FP.

## Results

### Transcriptome and differential expression analyses

**Fig. 1:**
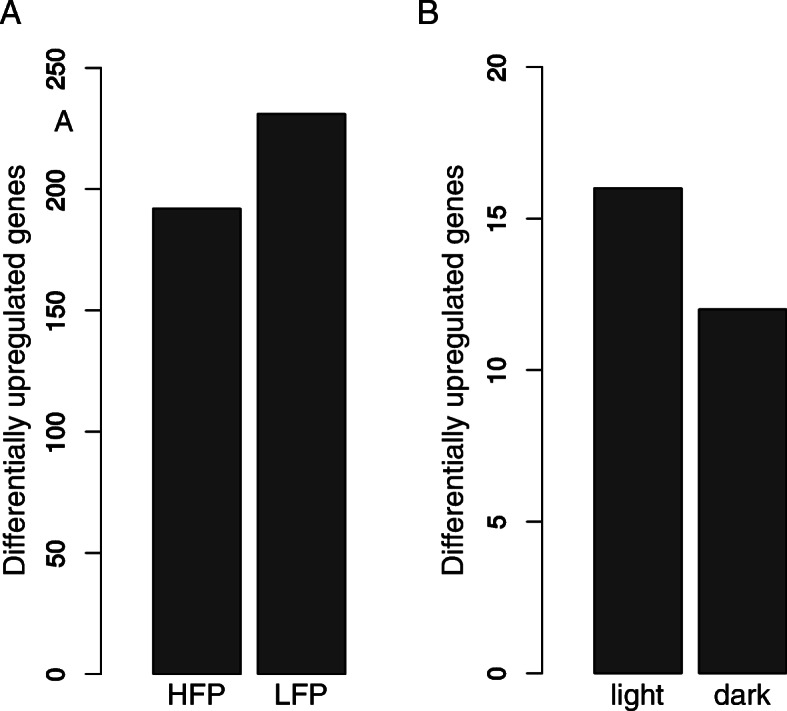
Number of significantly upregulated genes between (**a**) high feather peckers (HFP) and low feather peckers (LFP) as well as (**b**) laying hens after light stimulation (light) and before light stimulation (dark)

**Fig. 2:**
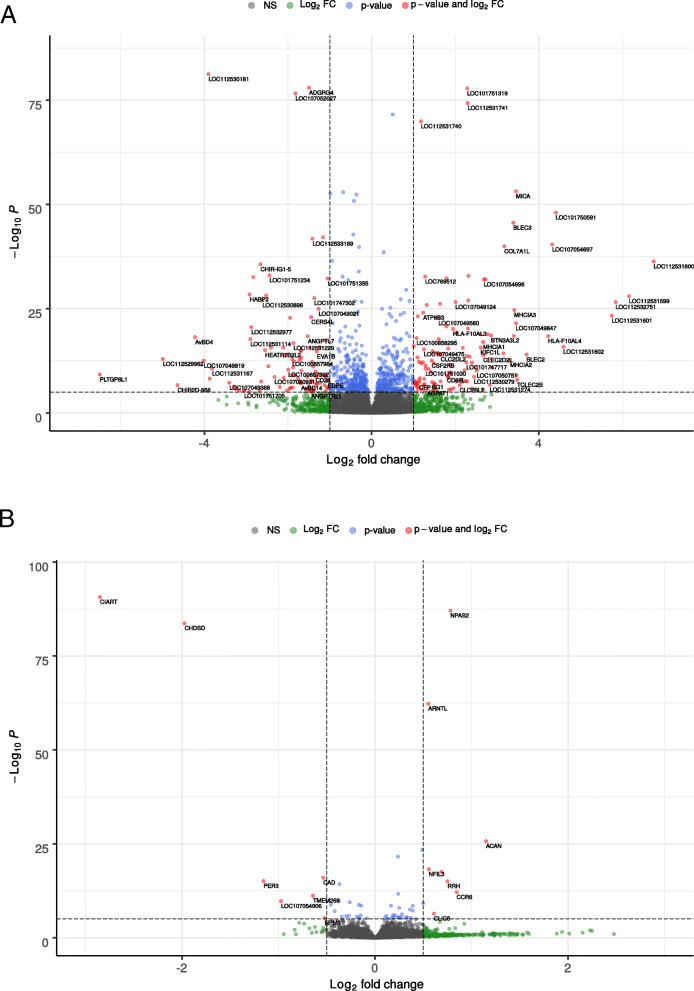
Volcano plots of DE genes between (**a**) high pecker and low pecker hens and between (**b**) chickens before and after light stimulation

**Fig. 3:**
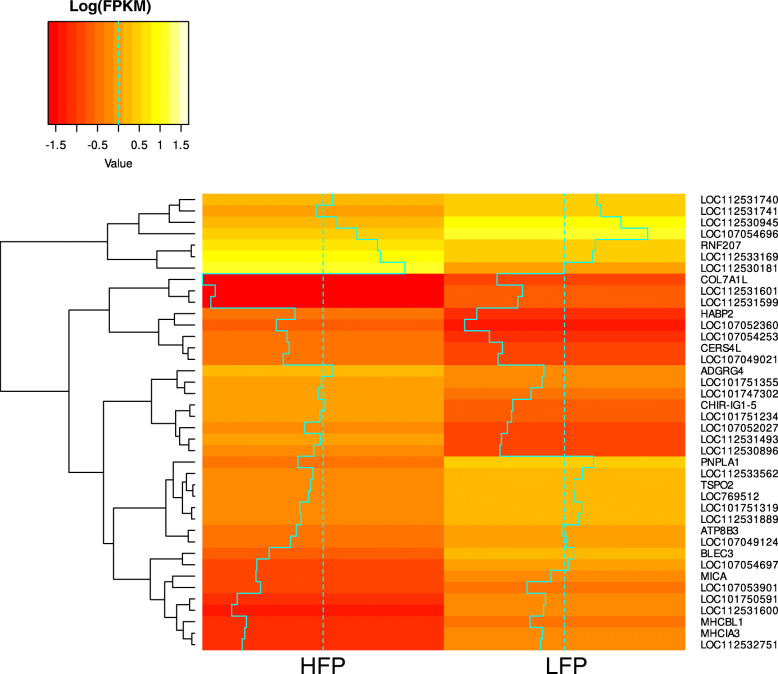
Heatmap of the top 40 DE genes between HFP and LFP hens with the lowest p_adj_. The average log(FPKM) is shown in the heatmap

The average number of reads per sample was 48,502,572 (SD = 6,816,183, MIN = 36,337,624, MAX = 69,407,025) with an average mapping efficiency of 98.4% after alignment to the reference genome with TopHat (detailed summary in Additional file 1). A total of 13,070 genes were expressed in the brains of the 48 analyzed animals (average FPKM > 1, FPKM values are summarized in Additional file 2). Differential expression analysis revealed that 423 genes were differentially expressed (DE) between high feather peckers (HFP) and low feather peckers (LFP) (abs. LogFC ≥1, p_adj_ < 0.01) (Figs. 1a and 2a). Among those, we found 305 genes of uncertain function (LOC symbols), of which 156 are classified as non-coding RNAs (ncRNAs). Light stimulation led to the discovery of 28 DE genes (abs. LogFC ≥0.5, p_adj_ < 0.01) (Figs. 1b and 2b) with no differences between HFP and LFP (summary of differential expression analysis in Additional file 3). The top 40 DE genes with the lowest p_adj_ between HFP and LFP are plotted in a Heatmap (Fig. 3). A table of putative enhancer RNAs (eRNAs; Table 1) contains the closest DE protein coding genes (abs. LogFC ≥0.5, p_adj_ < 0.01) to DE ncRNAs.

**Table 1:** Putative enhancer RNAs and their distance to possible targets

ncRNA	LogFC	p_adj_	closest gene	LogFC	p_adj_	distance (bp)
LOC112533590	2.199688474	0.003164539	AGWCL	0.701583764	0.000159023	84,768
LOC112532313	1.059839775	6.1822E-08	ARHGEF38	1.263617235	1.32471E-07	0
LOC107054701	2.235712776	0.000627778	BTN3A3L1	0.910991403	4.69011E-05	4116
LOC107053477	1.224734434	7.74962E-13	CD69L	1.802269534	0.000000001	45,910
LOC107055491	1.408150332	5.66615E-05	CD69L	1.802269534	0.000000001	55,660
LOC112531172	− 3.288998976	4.97887E-07	CHIR-B3	1.786356201	0.000385776	35,442
LOC107054530	−1.479256537	2.63879E-16	GLRA1	−0.97955118	4.18544E-06	0
LOC107054253	−1.945791606	1.66336E-23	MCTP2	0.500309061	7.24605E-10	159,951
LOC107054254	1.035893153	2.34176E-08	MCTP2	0.500309061	7.24605E-10	31,878
LOC112533579	−1.497091833	0.00226963	MHCIA7	2.301079795	5.8491E-21	398
LOC112533585	1.334870542	0.000257017	MHCIY	1.286449024	0.000638942	912
LOC112533594	1.666649682	0.000528436	MICA	3.453477244	7.27436E-54	0
LOC112533584	1.273414179	0.000170499	MICA	3.453477244	7.27436E-54	7938
LOC107051266	1.658081532	0.000148479	MICA	3.453477244	7.27436E-54	2538
LOC107057581	−2.429748947	0.003583672	MROH2B1	−2.057428188	1.87694E-14	5389
LOC107053142	−1.035600209	1.31726E-06	SH3YL1	−0.557882395	4.72211E-19	19,668
LOC107055047	−2.23006132	0.00045059	ST14	−0.987882765	2.7619E-53	0
LOC112531741	2.297070835	4.86832E-75	TPK1	0.503349743	2.69361E-72	109,006
LOC771247	1.36815265	8.59138E-05	VTG2	0.871553426	2.19279E-08	3143
LOC107053986	1.084007796	0.000152067	VTG2	0.871553426	2.19279E-08	0
LOC112533587	−1.591777998	0.000659726	ZNF226L	−0.662767846	5.7519E-08	6697

*AGWCL* antigen WC1.1, *ARHGEF38* Rho guanine nucleotide exchange factor 38, *BTN3A3L1* butyrophilin subfamily 3 member A3-like 1, *CD69L* CD69 molecule like, *CHIR-B3* immunoglobulin-like receptor CHIR-B3, *GLRA1* glycine receptor alpha 1, *MCTP2* multiple C2 and transmembrane domain containing 2, *MHCIA7* major histocompatibility complex, class I, A7, *MHCIY* MHC-like class I Y, *MICA* MHC class I polypeptide-related sequence A, *MROH2B1* maestro heat-like repeat family member 2B 1, *SH3YL1* SH3 and SYLF domain containing 1, *ST14* ST14 transmembrane serine protease matriptase, *TPK1* thiamin pyrophosphokinase 1, *VTG2* vitellogenin 2, *ZNF226L* zinc finger protein 226-like

Detailed information on putative eRNAs are available in in Additional file 4.

### Functional and pathway analyses

The results of the Gene Ontology (GO) and KEGG pathway analyses with DE genes between HFP and LFP hens with clusterProfiler [29] are shown in Table 2 (complete results in Additional file 5). Most of the GO terms assigned to the comparison of HFP and LFP lines are linked to synaptic functions and membrane channels when DE genes with an absolute LogFC < 0.5 were neglected. In particular, the DE genes *CHRNA2*, *CHRNA9*, *CHRNB3*, and *CHRNB4* were assigned to these GO terms.

**Table 2:** Gene set analysis of DE genes between HFP and LFP hens with clusterProfiler. The top 5 results of each category with a q value below 0.2 are shown

Category	ID	Description	q value
GO_BP	GO:0060079	excitatory postsynaptic potential	0.116362767
GO_BP	GO:0099565	chemical synaptic transmission, postsynaptic	0.116362767
GO_CC	GO:0005892	acetylcholine-gated channel complex	0.164248522
GO_CC	GO:0005887	integral component of plasma membrane	0.164248522
GO_CC	GO:0031226	intrinsic component of plasma membrane	0.164248522
GO_CC	GO:0000786	Nucleosome	0.164248522
GO_CC	GO:0044815	DNA packaging complex	0.164248522
GO_MF	GO:0005231	excitatory extracellular ligand-gated ion channel activity	0.006673199
GO_MF	GO:0022848	acetylcholine-gated cation-selective channel activity	0.006673199
GO_MF	GO:0022824	transmitter-gated ion channel activity	0.007209589
GO_MF	GO:0022835	transmitter-gated channel activity	0.007209589
GO_MF	GO:0098960	postsynaptic neurotransmitter receptor activity	0.007209589
KEGG	gga04514	Cell adhesion molecules (CAMs)	0.000581813
KEGG	gga05168	Herpes simplex virus 1 infection	0.011506023

Concerning the comparison of DE genes between animals before and after light stimulation (Table 3), the majority of IDs belonging to the category GO biological processes were linked to circadian rhythm. The DE genes *ARNTL*, *NFIL3*, *NPAS2*, and *PER2* are overrepresented in these GO terms. The category GO molecular function exclusively contains IDs related to gene expression.

**Table 3:** Gene set analysis of DE genes before and after light stimulation with clusterProfiler. The top 5 results of each category with a q value below 0.2 are shown

Category	ID	Description	q value
GO_BP	GO:0007623	circadian rhythm	1.91764E-05
GO_BP	GO:0048511	rhythmic process	6.88484E-05
GO_BP	GO:0032922	circadian regulation of gene expression	8.31201E-05
GO_BP	GO:0042752	regulation of circadian rhythm	0.022669043
GO_BP	GO:0045892	negative regulation of transcription, DNA-templated	0.076074608
GO_CC	GO:0005667	transcription factor complex	0.138038823
GO_MF	GO:0000980	RNA polymerase II distal enhancer sequence-specific DNA binding	0.006730784
GO_MF	GO:0001158	enhancer sequence-specific DNA binding	0.006730784
GO_MF	GO:0035326	enhancer binding	0.006730784
GO_MF	GO:0000976	transcription regulatory region sequence-specific DNA binding	0.00988553
GO_MF	GO:1990837	sequence-specific double-stranded DNA binding	0.00988553
KEGG	gga00250	Alanine, aspartate and glutamate metabolism	0.062575255
KEGG	gga00514	Other types of O-glycan biosynthesis	0.062575255
KEGG	gga04330	Notch signaling pathway	0.062575255
KEGG	gga00240	Pyrimidine metabolism	0.062575255
KEGG	gga04145	Phagosome	0.107115602

Geneset analysis with STRING [30] was performed on DE genes between HFP and LFP lines, where genes with an absolute LogFC < 1 were neglected (Table 4, complete results see Additional file 6). The majority of terms found in this analysis are linked to the immune system and immune response.

**Table 4:** DE genes between high feather peckers (HFP) and low feather peckers (LFP) were analyzed with STRING. The top 5 results of each category are shown

Category	ID	Description	FDR
InterPro	IPR007110	Immunoglobulin-like domain	0.00000275
InterPro	IPR036179	Immunoglobulin-like domain superfamily	0.00000331
InterPro	IPR013783	Immunoglobulin-like fold	0.0000264
InterPro	IPR003599	Immunoglobulin subtype	0.000068
InterPro	IPR033992	Natural killer cell receptor-like, C-type lectin-like domain	0.0000774
PFAM	PF13895	Immunoglobulin domain	0.0000256
PFAM	PF00047	Immunoglobulin domain	0.000076
PFAM	PF00059	Lectin C-type domain	0.0069
PFAM	PF00089	Trypsin	0.0093
PFAM	PF00147	Fibrinogen beta and gamma chains, C-terminal globular domain	0.0093
SMART	SM00409	Immunoglobulin	0.000000564
SMART	SM00034	C-type lectin (CTL) or carbohydrate-recognition domain (CRD)	0.0037
SMART	SM00020	Trypsin-like serine protease	0.0038
SMART	SM00186	Fibrinogen-related domains (FReDs)	0.0038
SMART	SM00336	B-Box-type zinc finger	0.05
UniProt KW	KW-0393	Immunoglobulin domain	0.0087
UniProt KW	KW-0732	Signal	0.0087
UniProt KW	KW-1133	Transmembrane helix	0.023

Analysis of DE genes before and after light stimulation with STRING (Table 5) led to the discovery of terms related to biological/circadian rhythm and gene expression as well.

**Table 5:** DE genes between chickens before and after light stimulation were analyzed with STRING. The top 5 results of each category are shown

Category	ID	Description	FDR
InterPro	IPR000014	PAS domain	0.00000421
InterPro	IPR035965	PAS domain superfamily	0.00000421
InterPro	IPR022728	Period circadian-like, C-terminal	0.00021
InterPro	IPR001067	Nuclear translocator	0.0012
InterPro	IPR013655	PAS fold-3	0.0016
PFAM	PF08447	PAS fold	0.000000543
PFAM	PF14598	PAS domain	0.000000543
PFAM	PF00989	PAS fold	0.0000712
PFAM	PF12114	Period protein 2/3C-terminal region	0.000078
PFAM	PF00001	7 transmembrane receptor (rhodopsin family)	0.0282
Reactome	GGA-373076	Class A/1 (Rhodopsin-like receptors)	0.0284
Reactome	GGA-418594	G alpha (i) signalling events	0.0284
SMART	SM00086	Motif C-terminal to PAS motifs (likely to contribute to PAS structural domain)	0.000000624
SMART	SM00091	PAS domain	0.000000624
SMART	SM00353	helix loop helix domain	0.0228
UniProt KW	KW-0090	Biological rhythms	0.00000116
UniProt KW	KW-0010	Activator	0.0159
UniProt KW	KW-0805	Transcription regulation	0.0251
UniProt KW	KW-0297	G-protein coupled receptor	0.0355

**Fig. 4:**
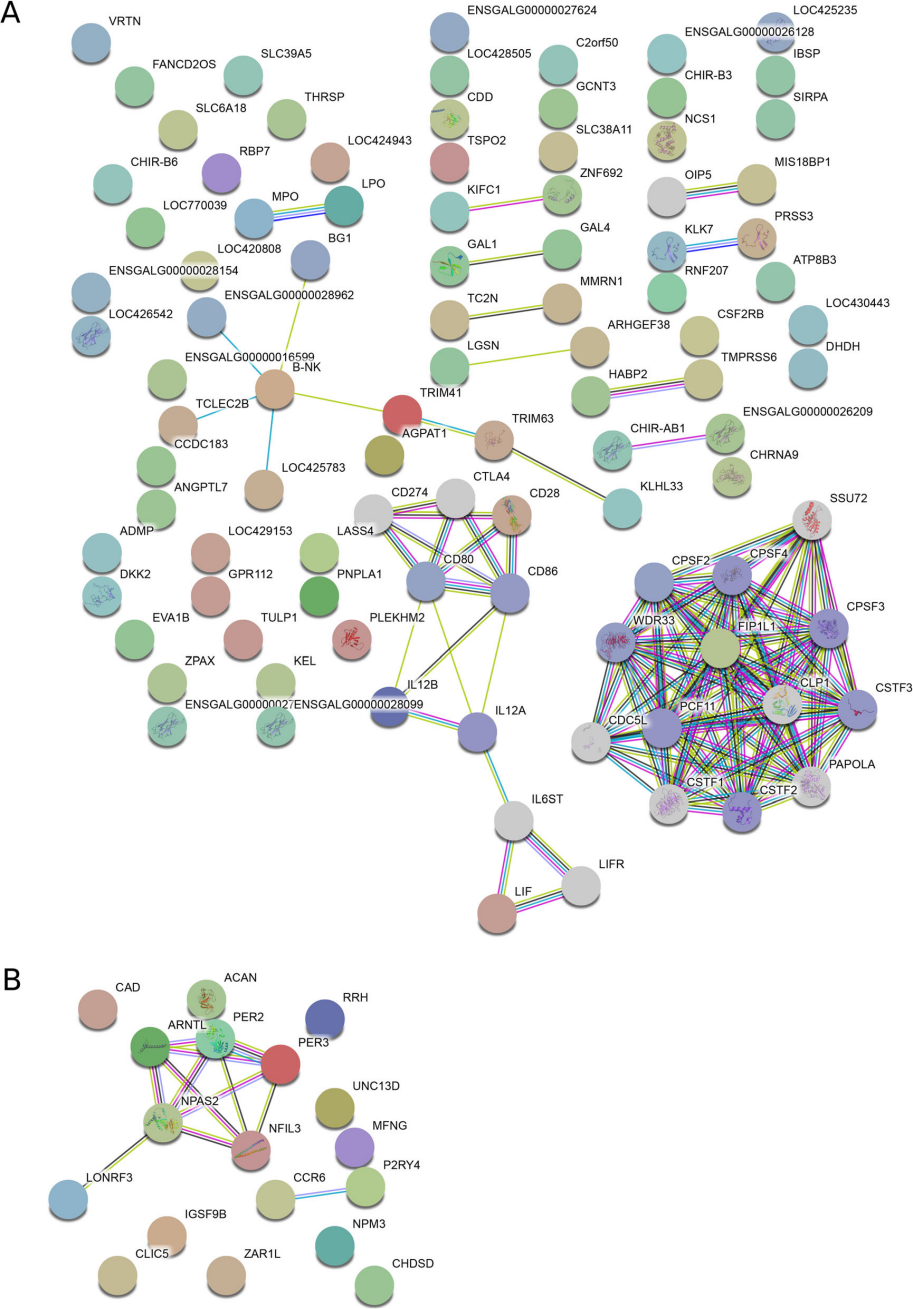
Protein interaction networks of DE genes between (**a**) high and low feather peckers and between (**b**) animals before and after light stimulation

Protein interaction network analysis with STRING revealed one large and one smaller interaction cluster in the comparison of HFP and HFP DE genes (Fig. 4a). The large cluster, consisting of the DE gene *FIP1L1* as well as *SSU72*, *CPSF2*, *CPSF3*, *CPSF4*, *WDR33*, *CDC5L*, *PCF11*, *CLP1*, *CSTF1*, *CSTF2*, *CSTF3*, and *PAPOLA*, exclusively contains genes, which are responsible for mRNA processing as well as mRNA binding. The slightly smaller cluster with fewer connections exclusively contains genes, which are associated with the immune system - T-cell proliferation and regulation in particular. The immune cluster consists of the DE genes *CD28* and *LIF* as well as *CD274*, *CTLA4*, *CD80*, *CD86*, *IL2B*, *IL12A*, *IL6ST*, and *LIFR*. Analysis of DE genes before and after light stimulation led to the discovery of one interaction cluster, which contains the DE genes *ARNTL*, *PER2*, *PER3*, *NFIL3*, *NPAS2*, and *LONRF3*, all of which, except LONFRF3, are core components of the circadian clock (Fig. 4b).

## Discussion

In this study, we compared 24 full-sib pairs of laying hens selected for HFP and LFP in a transcriptomics approach. Besides the line comparison, we assessed the transcriptional changes before and after light stimulation. To our knowledge, this is the first brain transcriptomic study in FP that used RNA-seq. Comparison with the few other available transcriptome studies revealed only little overlap, which might be due to the fact that these studies are microarray-based and applied different designs. Hughes and Buitenhuis [31] used a more complex phenotyping scheme distinguishing between severe and gentle FP, Brunberg et al. [23] particularly analyzed hypothalamic RNA and Wysocki et al. [22] considered much older birds. Some genes, however, that these authors reported were also found DE in the current study. Notably, Hughes and Buitenhuis reported the expression of *IRF2* (interferon regulatory factor 2) to be associated with severe FP This gene plays a major role in immune response [32] and might match our results with respect to immune related genes. In our study, however, we did not identify this gene, but found *IRF5* (p_adj_ = 0.0002), *IRF6* (p_adj_ = 1.17E-06), and *IRF8* (p_adj_ = 0.0002) to be DE between the lines. Wysocki et al. reported DE of genes related to monoamine signaling, namely *MAOA* (monoamine oxidase A) and *HTRR1B* (5-hydroxytryptamine (serotonin) receptor 1B). We were able to confirm *MAOA* (p_adj_ = 7.2E-05), but not *HTRR1B*. Instead, we found DE of *HTR1A* (p_adj_ = 0.04), *HTR1E* (p_adj_ = 0.001) and *HTR6* (p_adj_ = 2.34E-05).

Most of the GO terms found enriched in the current study in genes DE between HFP and LFP lines are linked to synaptic function when a LogFC threshold of 0.5 is used. This, in particular, is due to the DE genes *CHRNA2*, *CHRNA9*, *CHRNB3*, and *CHRNB4* coding for subunits of nicotinic acetylcholine receptors (nAchR). Most other studies found serotonergic or dopaminergic signaling to be involved in FP [18] and we recently reported a possible involvement of GABAergic signaling based on genomic studies in the very same population as used in the current study [21, 25]. Cholinergic signaling has not been implicated in FP so far, but is linked to both monoamine as well as GABAergic signaling and is implicated in certain psychiatric disorders [33]. In particular, α7 nAChRs have a crucial role in the dysfunction of cortical parvalbumin-positive GABAergic neurons, as seen in schizophrenia [34]. The activation of the dopamine system by stimulating α7 nAChRs is crucial in drug dependence and withdrawal effects including motor function and rewarding properties of drug intake [35, 36]. In turn, it was shown that α7 nAChR blockade in mice can reduce anxiety-like behaviors as a consequence of nicotine withdrawal [37]. Nicotinic α4β2 receptors, on the other hand, have been implicated in obsessive-compulsive disorder and it has been shown that positive allosteric modulation of these receptors attenuated the obsessive behavior [38]. The actual behavioral effects of nAChRs are mediated by dopamine and glutamate and ultimately by GABAergic neurons [39, 40]. Very recently, it was shown in rats that β2 nAChRs on dopamine neurons in the ventral tegmental area mediate nicotine’s conditioned aversive effects, while those on GABA neurons mediate the conditioned rewarding effects [41]. In our study, we did not find the same subunits as described above to be differentially expressed, but all other studies so far have been conducted in humans, mice or rats and little is known in chicken. Thus, the findings might possibly point to a model of FP, in which cholinergic, monoaminergic and GABAergic signaling in the central nervous system are interacting. If this holds true, it seems likely that nAChRs might be on top of this signaling hierarchy. In a second gene set analysis, we used a more stringent set of genes by setting a LogFC threshold of 1.0. Interestingly this led to a shift in enriched terms compared to the aforementioned analysis to immunoglobulin-associated genes. We detected a protein interaction cluster consisting of 10 genes (the DE genes *CD28* and *LIF* as well as *CD274*, *CTLA4*, *CD80*, *CD86*, *IL2B*, *IL12A*, *IL6ST*, and *LIFR*), all of which are linked to T-cell proliferation and regulation (Fig. 4a). In a recent study on post-mortem human brains, an increased number of T-cells was found in the brains of schizophrenia patients [42]. A probable mechanism, by which an increase in the number of T cells could influence the propensity to FP has been discovered in a murine cell culture system. Mashimo et al. demonstrated that T cells are able to synthesize acetylcholine (ACh) but also respond to it via nAChRs by increased cell proliferation and increased Ca^2+^ levels [43]. We propose a mechanism in which an overrepresentation of T cells leads to excess ACh production in the brain that has two major effects: (i) nAChR activation in neurons and (ii) further increase of T cell proliferation in a positive feedback loop. Since the immune-modulating genes show a higher LogFC compared to genes encoding nAChR subunits, we hypothesize that the dysregulation of T cell proliferation and activation is the initial cause of this neuropsychiatric phenotype. A recent extensive transcriptome study in humans suffering from autism spectrum disorder, schizophrenia, and bipolar disorder identified pathways related to the immune system and to transmembrane transporters/receptors to be differentially regulated in comparison to healthy individuals [44], which indicates that similar mechanisms are responsible for neuropsychiatric disorders between species. Interestingly these researchers found a large amount of DE ncRNAs between patients and controls, which they termed “psychiatric ncRNAs”. With 36.9%, ncRNAs make up a large part of significant DE transcripts between HFP and LFP hens. Gandal et al. already proposed these putative “psychiatric ncRNAs” to be responsible for transcriptome dysregulation by regulating local splicing events. This might be connected to the observation that HFP animals show a globally reduced variance in gene expression [31].

However, comparison of cDNA sequences of the DE ncRNAs identified in this study with the “psychiatric ncRNAs” from Gandal et al. with discontiguous megablast [45] showed sequence homology between only one pair of ncRNAs: The human ncRNA ENSG00000234773, which is a novel zinc finger protein pseudogene, is partially homologous to LOC112533587. Due to the taxonomic distance, the huge difference in genome size, and the fact that ncRNAs are less evolutionary conserved than protein coding genes [46], we did not expect to find homologous ncRNAs between *Homo sapiens* and *Gallus gallus*. By finding the closest DE expressed protein coding genes to DE ncRNAs between HFP and LFP chickens we were able to identify 21 putative eRNAs (Table 1). Among possible targets of these eRNA candidates are *ARHGEF38* and *SH3YL1*, which have been linked to bipolar disorder and suicide [47, 48], *GLRA1* and *MCTP2* were found to be associated with schizophrenia [49, 50], and *CHIR-B3*, *MHCIA7*, *MHCIY* as well as *MICA* are genes involved in the immune system [51]. Thus, further experiments, like overexpression studies, will help to clarify the involvement of particular ncRNAs in FP. The second objective of this study was to clarify if HFP and LFP lines respond differently to light stimuli. Shi et al. demonstrated that red light and low light intensity was shown to reduce FP behavior during the laying period [28]. Light stimulation led to DE of five genes, which are core components of circadian clock (Fig. 4b) but there were no differences detectable in the light response between HFP and LFP.

Whereas in the current study whole brains where analyzed, it could be especially useful to restrict analyses to particular brain regions e.g. arcopallium or caudocentral nidopallium where differences in the serotonin or dopamine metabolism between LFP and HFP were found [52, 53]. Furthermore, analyses should be conducted in a larger number of birds and accounting for the actual phenotype, i.e. comparing performers against non-performer. Here, we compared divergently selected lines, which might also differ in other traits than FP [54]. Although the lines were initially derived from the same founder population and selection was solely based on the estimated breeding value for feather pecking, genetic drift may have caused allele frequency differences between the lines [25]. Here, actual behavior was highly correlated with line with on average 1.6 bouts per bird in LFP and 12.7 bouts per bird in HFP animals during the standardized observation time. A phenotype-based approach would have resulted in an almost identical group assignment. Futures studies should aim to validate the results in a purely phenotype-based approach using a single and unselected field population. This is, however, not trivial, because the occurrence of FP cannot be reliably predicted in commercial flocks and phenotyping is challenging under field conditions, which is the reason for many studies being conducted in selection lines.

## Conclusions

The analysis of whole-brain transcriptomes in 24 full-sib pairs of hens from two White Leghorn layer strains divergently selected for FP behavior revealed differentially expressed genes and pathways related to cholinergic signaling and immune response. Thus, we hypothesize an involvement of these genes and pathways in the development of FP. Previous studies unambiguously identified monoamine signaling as the key component in FP behavior, but also pointed to an involvement of GABAergic signaling, immune response and the gut microbiota. Our results provide a first building block for an integrated model of FP. In this model, the gut microbiota might be involved in the interaction between the peripheral and the central serotonergic system as well as in the modulation of the immune response. Via the latter route, there could be a connection with cholinergic signaling, which in turn projects into monoamine signaling, directly or via GABA or glutamate signaling. In coming studies, the current results have to be validated using alternative approaches and appropriate functional studies need to be designed and conducted to further challenge and develop this model.

## Methods

### Experimental animals and sample collection

**Fig. 5:**
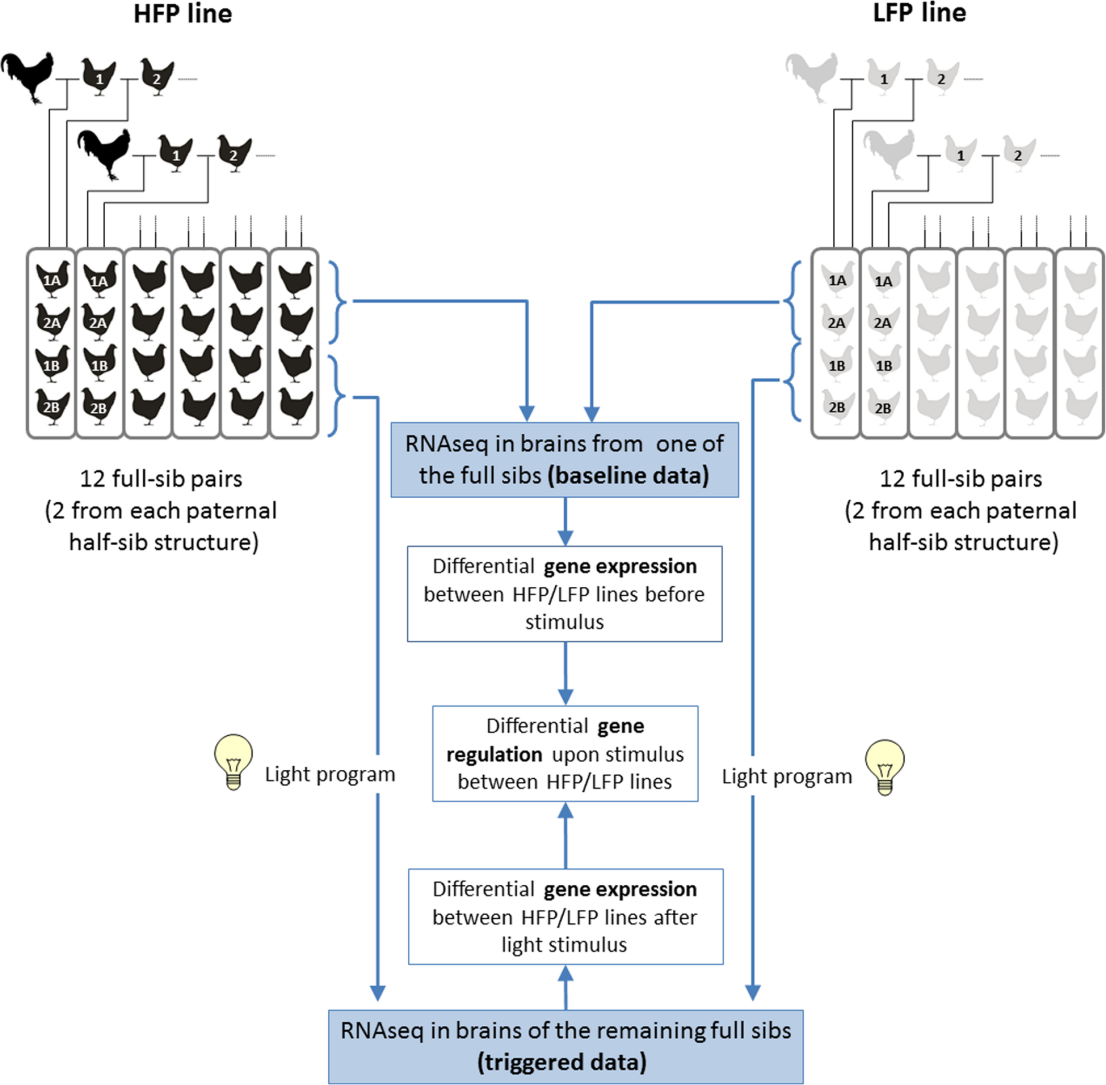
Experimental design of the study

Experiments were conducted in White Leghorn layer strains divergently selected for FP behavior [2, 55]. Strains were developed from a single founder population and selection over 15 generations was solely based on estimated breeding values for feather pecking [17]. These lines were created and are maintained at the Hohenheim University and neither commercially obtained nor from a private source. Details on hatching, rearing, and husbandry have previously been described in detail [25, 56]. A total of 48 hens comprising 12 full-sib pairs from each strain (high feather peckers, HFP vs. low feather peckers, LFP) were phenotyped according to established protocols at 27 weeks of age [25]. Briefly, animals were observed on four consecutive days in sessions of 20 min by at least six different experienced observers. FP was defined as non-aggressive severe pecks or pulls directed to the plumage of conspecifics [57]. A series of pecks delivered in a short sequence without changing the behavior was recorded as a single occurrence called a bout per bird. Subsequently, birds were kept under low light conditions to prevent FP (Fig. 5). One bird from each full-sib pair was then sacrificed and brains were immediately collected for RNA isolation. Chickens were CO_2_-stunned and sacrificed by ventral neck cutting. The remaining birds were kept under increased light intensity (≥100 lx) for several hours until they clearly showed FP and were then sacrificed as well and brains were collected for RNA isolation. All brain samples were kept in RNA later and frozen at − 80 °C until further processing.

### RNA isolation

Whole chicken brains were pulverized with a Retsch mixer mill MM400 in liquid nitrogen at a frequency of 30 Hz for 1 min and immediately frozen at − 80 °C. For RNA isolation 900 μl Qiazol (QIAGEN), 5 μl DX reagent and 20–30 ceramic beads were added to10–30 mg of pulverized brain. The mixture was placed in a Bead Ruptor (Omin Inc.) and homogenized with the following program: 4.5 m/sec, 3 times 15 s, 10s dwell. After 5 min incubation at room temperature 100 μl gDNA Eliminator solution (QIAGEN) was added and the sample was vortexed for 15 s. After addition of 180 μl chloroform the sample was vortexed for 15 s and incubated at room temperature for 3 min. Sample was centrifuged at 12,000 g and 4 °C for 20 min and aqueous phase was transferred to a fresh reaction tube. An equal amount of 70% ethanol was added to the sample and vortexed for 15 s. RNA was purified using the RNeasy Plus Universal Mini Kit (QIAGEN) according to the manufacturer’s protocol and stored at − 80 °C.

### NGS library preparation and sequencing

Input total RNA was visualized on a TapeStation 4200 (Agilent) and all but three samples showed excellent RNA quality with RIN-scores > 8 (three samples with RIN scores of 6.8, 6.9 and 7.7 respectively). Samples were quantified on a Qubit 2.0 Fluorometer (ThermoFischer) and 500 ng of total RNA was used as input for the TruSeq stranded mRNA library kit (Illumina) following the manufacturers manual. Resulting libraries showed a fragment size distribution of around 300 bp and were 2 × 75 bp paired-end. Sequencing was conducted on the HiSeq 4000 (Illumina) with 10 samples / lane.

### Transcriptome analyses

Raw sequencing reads were processed with Trimmomatic version 0.36 [58] for adapter removal, trimming of low quality base calls, and removal of low-quality reads. Trimmomatic was used with the settings: PE -phred33 LEADING:3 TRAILING:3 SLIDINGWINDOW:4:15 MINLEN:36. Read pairs were discarded if one read did not survive quality control. Trimmed reads were aligned to chicken genome version GCF_000002315.5 (see Availability of data and materials) using TopHat version 2.1.0 [59] with the settings --no-novel-juncs --min-isoform-fraction 0.0 --min-anchor-length 3 -r 192 and GCF_000002315.5.gff (see Availability of data and materials) as known transcript file. Genomic features were extracted from the general feature format file and grouped with the R package GenomicFeatures (Version 1.40.0) [60]. summarizeOverlaps from the R package GenomicAlignments (Version 1.24.0) was used to count exon spanning reads [60]. Differential expression between experimental groups was analyzed with DESeq2 (Version 1.28.1) [61]. Volcano plots of differential expression analyses were created with the R package EnhancedVolcano (Kevin Blighe, Sharmila Rana and Myles Lewis (2019). EnhancedVolcano (Version 1.6.0). All R packages were obtained with Bioconductor version 3.11.

### Identification of eRNA candidates

One BED file was created containing DE ncRNAs and one BED file containing all protein coding genes annotated in chicken genome version GCF_000002315.5 (see Availability of data and materials). The closest coding gene to each respective ncRNA was identified with BEDtools (Version 2.27.1) [62].

### Functional analyses

Gene set analyses were conducted using the R package clusterProfiler (Version 3.16.0) [29] along with the chicken genome annotation org.Gg.eg.db (Version 3.11.4) [63] and using default settings in terms of gene set size and *p*-value correction. Genes differentially expressed with an absolute LogFC ≥0.5 were analyzed for enrichment in GO terms and KEGG pathways against the background of all expressed genes in our data set. For the gene set analysis with STRING (Version 11.0) [8], DE genes between HFP and LFP laying lines with an absolute LogFC ≥1 and an adjusted *p*-value below 0.01 were used. Protein interaction networks for these genes were also computed with STIRNG using the following settings: evidence-based coloring of network edges, inclusion of all interaction sources, medium required interaction score 0.4, max number of interactions to show 1st shell: no more than 10 interactions, 2nd shell: no more than 10 interactions. For DE genes before and after light stimulation LogFC threshold was 0.5. Settings for protein interaction networks were as follows: evidence based coloring of network edges, inclusion of all interaction sources, medium required interaction score 0.4, max number of interactions to show 1st shell: none / query proteins only, 2nd shell: none.

## Supplementary information

**Additional file 1.** Transcriptome alignment summary.

**Additional file 2.** FPKM values.

**Additional file 3.** Results of differential expression analyses.

**Additional file 4.** Detailed information on eRNAs.

**Additional file 5.** Results of clusterProfile analyses.

**Additional file 6.** Results of STRING analyses.

## Data Availability

RNA-seq reads were mapped to chicken genome version GCF_000002315.5 (RefSeq assembly, https://ftp.ncbi.nlm.nih.gov/genomes/all/GCF/000/002/315/GCF_000002315.5_GRCg6a/GCF_000002315.5_GRCg6a_genomic.fna.gz). Gene features were derived from GCF_000002315.5.gff (https://ftp.ncbi.nlm.nih.gov/genomes/all/GCF/000/002/315/GCF_000002315.5_GRCg6a/GCF_000002315.5_GRCg6a_genomic.gff.gz). The datasets used and/or analysed during the current study are available from the corresponding author on reasonable request. The raw sequencing data of the transcriptome experiments including phenotype and treatment information are accessible via BioProject ID PRJNA656654.

## Abbreviations

ACh: Acetylcholine
DE: Differentially expressed
eRNAs: Enhancer RNAs
FP: Feather pecking
FPKM: Fragments per kilobase of exon model per million reads mapped
GO: Gene ontology
HFP: High feather pecker
LFP: Low feather pecker
LogFC: Log fold change
nAchR: Nicotinic acetylcholine receptors
ncRNAs: Non-coding RNAs
p_adj_: Adjustet *p*-value
RNA-seq: RNA-sequencing

## References

[1] Rodenburg TB, Buitenhuis AJ, Ask B, Uitdehaag KA, Koene P, van der Poel JJ, Bovenhuis H (2003). Heritability of feather pecking and open-field response of laying hens at two different ages. Poult Sci.

[2] Grams V, Wellmann R, Preuß S, Grashorn MA, Kjaer JB, Bessei W, Bennewitz J (2015). Genetic parameters and signatures of selection in two divergent laying hen lines selected for feather pecking behaviour. Genet Sel Evol.

[3] Kjaer JB, Sørensen P (1997). Feather pecking behaviour in white leghorns, a genetic study. Br Poult Sci.

[4] Blokhuis HJ (1986). Feather-pecking in poultry: its relation with ground-pecking. Appl Anim Behav Sci.

[5] Wechsler B, Huber-Eicher B (1998). The effect of foraging material and perch height on feather pecking and feather damage in laying hens. Appl Anim Behav Sci.

[6] Kjaer JB (2009). Feather pecking in domestic fowl is genetically related to locomotor activity levels: implications for a hyperactivity disorder model of feather pecking. Behav Genet.

[7] Lutz V, Kjaer JB, Iffland H, Rodehutscord M, Bessei W, Bennewitz J (2016). Quantitative genetic analysis of causal relationships among feather pecking, feather eating, and general locomotor activity in laying hens using structural equation models. Poult Sci.

[8] Bessei W, Kjaer JB, Poultry Research Foundation (2005). Feather pecking in layers-state of research and implications.

[9] Meyer B, Bessei W, Bessei AW, Vahjen W, Zentek J, Harlander-Matauschek A (2012). Dietary inclusion of feathers affects intestinal microbiota and microbial metabolites in growing Leghorn-type chickens. Poult Sci.

[10] Kriegseis I, Bessei W, Meyer B, Zentek J, Würbel H, Harlander-Matauschek A (2012). Feather-pecking response of laying hens to feather and cellulose-based rations fed during rearing. Poult Sci.

[11] Birkl P, Bharwani A, Kjaer JB, Kunze W, McBride P, Forsythe P, Harlander-Matauschek A (2018). Differences in cecal microbiome of selected high and low feather-pecking laying hens. Poult Sci.

[12] van der Eijk JAJ, de Vries H, Kjaer JB, Naguib M, Kemp B, Smidt H (2019). Differences in gut microbiota composition of laying hen lines divergently selected on feather pecking. Poult Sci.

[13] Meyer B, Zentek J, Harlander-Matauschek A (2013). Differences in intestinal microbial metabolites in laying hens with high and low levels of repetitive feather-pecking behavior. Physiol Behav.

[14] Rodenburg TB, van Krimpen MM, de Jong IC, de Haas EN, Kops MS, Riedstra BJ (2013). The prevention and control of feather pecking in laying hens: identifying the underlying principles. Worlds Poult Sci J.

[15] Kops MS, Kjaer JB, Güntürkün O, Westphal KGC, Korte-Bouws GAH, Olivier B (2014). Serotonin release in the caudal nidopallium of adult laying hens genetically selected for high and low feather pecking behavior: an in vivo microdialysis study. Behav Brain Res.

[16] van Hierden YM, de Boer SF, Koolhaas JM, Korte SM (2004). The control of feather pecking by serotonin. Behav Neurosci.

[17] Coates MD, Tekin I, Vrana KE, Mawe GM (2017). Review article: the many potential roles of intestinal serotonin (5-hydroxytryptamine, 5-HT) signalling in inflammatory bowel disease. Aliment Pharmacol Ther.

[18] de Haas EN, van der Eijk JAJ (2018). Where in the serotonergic system does it go wrong? Unravelling the route by which the serotonergic system affects feather pecking in chickens. Neurosci Biobehav Rev.

[19] Parmentier HK, Rodenburg TB, de Vries Reilingh G, Beerda B, Kemp B (2009). Does enhancement of specific immune responses predispose laying hens for feather pecking?. Poult Sci.

[20] van der Eijk JAJ, Verwoolde MB, de Vries Reilingh G, Jansen CA, Rodenburg TB, Lammers A (2019). Chicken lines divergently selected on feather pecking differ in immune characteristics. Physiol Behav.

[21] Lutz V, Stratz P, Preuß S, Tetens J, Grashorn MA, Bessei W, Bennewitz J (2017). A genome-wide association study in a large F2-cross of laying hens reveals novel genomic regions associated with feather pecking and aggressive pecking behavior. Genet Sel Evol.

[22] Wysocki M, Preuss S, Stratz P, Bennewitz J (2013). Investigating gene expression differences in two chicken groups with variable propensity to feather pecking. Anim Genet.

[23] Brunberg E, Jensen P, Isaksson A, Keeling L (2011). Feather pecking behavior in laying hens: hypothalamic gene expression in birds performing and receiving pecks. Poult Sci.

[24] Flisikowski K, Schwarzenbacher H, Wysocki M, Weigend S, Preisinger R, Kjaer JB, Fries R (2009). Variation in neighbouring genes of the dopaminergic and serotonergic systems affects feather pecking behaviour of laying hens. Anim Genet.

[25] Iffland H, Wellmann R, Schmid M, Preuß S, Tetens J, Bessei W, Bennewitz J (2020). Genomewide Mapping of Selection Signatures and Genes for Extreme Feather Pecking in Two Divergently Selected Laying Hen Lines. Animals (Basel).

[26] Biscarini F, Bovenhuis H, van der Poel J, Rodenburg TB, Jungerius AP, van Arendonk JAM (2010). Across-line SNP association study for direct and associative effects on feather damage in laying hens. Behav Genet.

[27] Riber AB, Guzman DA. Effects of dark brooders on behavior and fearfulness in layers. Animals (Basel). 2016. 10.3390/ani6010003. 10.3390/ani6010003 PMC4730120 26751482

[28] Shi H, Li B, Tong Q, Zheng W, Zeng D, Feng G. Effects of LED light color and intensity on feather pecking and fear responses of layer breeders in natural mating Colony cages. Animals (Basel). 2019. 10.3390/ani9100814. 10.3390/ani9100814 PMC6826393 31623071

[29] Yu G, Wang L-G, Han Y, He Q-Y (2012). clusterProfiler: an R package for comparing biological themes among gene clusters. OMICS.

[30] Szklarczyk D, Gable AL, Lyon D, Junge A, Wyder S, Huerta-Cepas J (2019). STRING v11: protein-protein association networks with increased coverage, supporting functional discovery in genome-wide experimental datasets. Nucleic Acids Res.

[31] Hughes AL, Buitenhuis AJ (2010). Reduced variance of gene expression at numerous loci in a population of chickens selected for high feather pecking. Poult Sci.

[32] Chae M, Kim K, Park S-M, Jang I-S, Seo T, Kim D-M (2008). IRF-2 regulates NF-kappaB activity by modulating the subcellular localization of NF-kappaB. Biochem Biophys Res Commun.

[33] Kim J, Yang JH, Ryu IS, Sohn S, Kim S, Choe ES. Interactions of Glutamatergic neurotransmission and brain-derived Neurotrophic factor in the regulation of behaviors after nicotine administration. Int J Mol Sci. 2019. 10.3390/ijms20122943. 10.3390/ijms20122943 PMC6627482 31208140

[34] Lin H, Hsu F-C, Baumann BH, Coulter DA, Anderson SA, Lynch DR (2014). Cortical parvalbumin GABAergic deficits with α7 nicotinic acetylcholine receptor deletion: implications for schizophrenia. Mol Cell Neurosci.

[35] Livingstone PD, Wonnacott S (2009). Nicotinic acetylcholine receptors and the ascending dopamine pathways. Biochem Pharmacol.

[36] Dani JA (2003). Roles of dopamine signaling in nicotine addiction. Mol Psychiatry.

[37] Jackson A, Papke RL, Damaj MI (2018). Pharmacological modulation of the \alpha7 nicotinic acetylcholine receptor in a mouse model of mecamylamine-precipitated nicotine withdrawal. Psychopharmacology.

[38] Mitra S, Mucha M, Khatri SN, Glenon R, Schulte MK, Bult-Ito A (2016). Attenuation of compulsive-like behavior through positive allosteric modulation of α4β2 nicotinic acetylcholine receptors in non-induced compulsive-like mice. Front Behav Neurosci.

[39] Ryu IS, Kim J, Seo SY, Yang JH, Oh JH, Lee DK, et al. Repeated Administration of Cigarette Smoke Condensate Increases Glutamate Levels and Behavioral Sensitization. Front Behav Neurosci. 2018. 10.3389/fnbeh.2018.00047. 10.3389/fnbeh.2018.00047 PMC5864865 29615877

[40] Ryu IS, Kim J, Seo SY, Yang JH, Oh JH, Lee DK (2017). Behavioral changes after nicotine challenge are associated with α7 nicotinic acetylcholine receptor-stimulated glutamate release in the rat dorsal striatum. Sci Rep.

[41] Grieder TE, Besson M, Maal-Bared G, Pons S, Maskos U, van der Kooy D (2019). β2* nAChRs on VTA dopamine and GABA neurons separately mediate nicotine aversion and reward. Proc Natl Acad Sci U S A.

[42] Sneeboer MAM, van Mierlo HC, Stotijn E, MacIntyre DJ, Smith C, Kahn RS, et al. Increased number of T-lymphocytes in post-mortem brain tissue of patients with schizophrenia. Schizophr Res. 2019. 10.1016/j.schres.2019.10.032. 10.1016/j.schres.2019.10.032 31676171

[43] Mashimo M, Iwasaki Y, Inoue S, Saito S, Kawashima K, Fujii T (2017). Acetylcholine released from T cells regulates intracellular Ca2+, IL-2 secretion and T cell proliferation through nicotinic acetylcholine receptor. Life Sci.

[44] Gandal MJ, Zhang P, Hadjimichael E, Walker RL, Chen C, Liu S, et al. Transcriptome-wide isoform-level dysregulation in ASD, schizophrenia, and bipolar disorder. Science. 2018. 10.1126/science.aat8127. 10.1126/science.aat8127 PMC6443102 30545856

[45] Altschul SF, Madden TL, Schäffer AA, Zhang J, Zhang Z, Miller W, Lipman DJ (1997). Gapped BLAST and PSI-BLAST: a new generation of protein database search programs. Nucleic Acids Res.

[46] Qu Z, Adelson DL (2012). Evolutionary conservation and functional roles of ncRNA. Front Genet.

[47] Gaine ME, Seifuddin F, Sabunciyan S, Lee RS, Benke KS, Monson ET (2019). Differentially methylated regions in bipolar disorder and suicide. Am J Med Genet B Neuropsychiatr Genet.

[48] Gaynor SC, Monson ET, Gaine ME, Chimenti MS, Reichman RD, Parsons M (2020). Male-specific association of the 2p25 region with suicide attempt in bipolar disorder. J Psychiatr Res.

[49] Vora AK, Fisher AM, New AS, Hazlett EA, McNamara M, Yuan Q (2017). Dimensional traits of Schizotypy associated with Glycine receptor GLRA1 polymorphism: an exploratory candidate-Gene Association study. J Personal Disord.

[50] Djurovic S, Le Hellard S, Kähler AK, Jönsson EG, Agartz I, Steen VM (2009). Association of MCTP2 gene variants with schizophrenia in three independent samples of Scandinavian origin (SCOPE). Psychiatry Res.

[51] Maenaka K, Jones EY (1999). MHC superfamily structure and the immune system. Curr Opin Struct Biol.

[52] Kops MS, de Haas EN, Rodenburg TB, Ellen ED, Korte-Bouws GAH, Olivier B (2013). Effects of feather pecking phenotype (severe feather peckers, victims and non-peckers) on serotonergic and dopaminergic activity in four brain areas of laying hens (Gallus gallus domesticus). Physiol Behav.

[53] Kops MS, Kjaer JB, Güntürkün O, Westphal KGC, Korte-Bouws GAH, Olivier B (2017). Brain monoamine levels and behaviour of young and adult chickens genetically selected on feather pecking. Behav Brain Res.

[54] Kjaer JB, Sørensen P, Su G (2001). Divergent selection on feather pecking behaviour in laying hens (Gallus gallus domesticus). Appl Anim Behav Sci.

[55] Bessei W, Bauhaus H, Bögelein S (2013). The effect of selection for high and low feather pecking on aggression – related behaviours of laying hens. Archiv für Geflügelkunde.

[56] Bennewitz J, Bögelein S, Stratz P, Rodehutscord M, Piepho HP, Kjaer JB, Bessei W (2014). Genetic parameters for feather pecking and aggressive behavior in a large F2-cross of laying hens using generalized linear mixed models. Poult Sci.

[57] Grams V, Bögelein S, Grashorn MA, Bessei W, Bennewitz J (2015). Quantitative genetic analysis of traits related to fear and feather pecking in laying hens. Behav Genet.

[58] Bolger AM, Lohse M, Usadel B (2014). Trimmomatic: a flexible trimmer for Illumina sequence data. Bioinformatics.

[59] Trapnell C, Pachter L, Salzberg SL (2009). TopHat: discovering splice junctions with RNA-Seq. Bioinformatics..

[60] Lawrence M, Huber W, Pagès H, Aboyoun P, Carlson M, Gentleman R (2013). Software for computing and annotating genomic ranges. PLoS Comput Biol.

[61] Love MI, Huber W, Anders S (2014). Moderated estimation of fold change and dispersion for RNA-seq data with DESeq2. Genome Biol.

[62] Quinlan AR, Hall IM (2010). BEDTools: a flexible suite of utilities for comparing genomic features. Bioinformatics.

[63] Carlson M (2019). Genome wide annotation for Chicken: R package.

